# Gigahertz Cutoff Frequencies and High Gain in Graphene-Based Hot-Electron Transistor Enabled by Material Engineering

**DOI:** 10.1021/acsami.6c04685

**Published:** 2026-06-20

**Authors:** Carsten Strobel, André Heinzig, Andre Hiess, Martin Knaut, Md Tarik Hossain, Andrey Turchanin, Tilo Meister, Frank Ellinger, Jens Trommer, Viktor Havel, Thomas Mikolajick

**Affiliations:** † Institute of Semiconductors and Microsystems, Chair of Nanoelectronics, 123108 Technische Universität Dresden, Nöthnitzer Straße 64, 01187 Dresden, Germany; ‡ 9378 Friedrich-Schiller-Universität Jena, Lessingstraße 10, 07743 Jena, Germany; § Chair of Circuit Design and Network Theory, Technische Universität Dresden, Helmholtzstraße 18, 01069 Dresden, Germany; ∥ 435872 NaMLab gGmbH, Nöthnitzer Str. 64 a, 01187 Dresden, Germany

**Keywords:** transistor, graphene, 2D materials, hot-electron transistor, cutoff frequency, current gain, gigahertz

## Abstract

Graphene (Gr)-based hot-electron transistors (GHETs) offer high potential for high-frequency applications due to the extremely thin nature of graphene as a base material. For the first time we present GHETs with excellent DC device characteristics and gigahertz operation achieved by an optimization of the emitter-base (E/B) composition. The optimized E/B composition enables more efficient injection of hot electrons into the base, thereby improving charge transport and reducing scattering losses. As a result, a record-high measured common-emitter current gain beta of 42 was achieved using a SiO_2_/Gr E/B structure. Moreover, when using a MoS_2_/Gr E/B junction the maximum output current is increased to record values of approximately 2000 A/cm^2^, representing a significant improvement in performance over previous devices. Furthermore, cutoff frequencies of close to 1 GHz are determined for nonoptimized SiO_2_/Gr-based devices. The experimentally observed device characteristics are very promising for future high-speed nanoelectronics.

## Graphical abstract

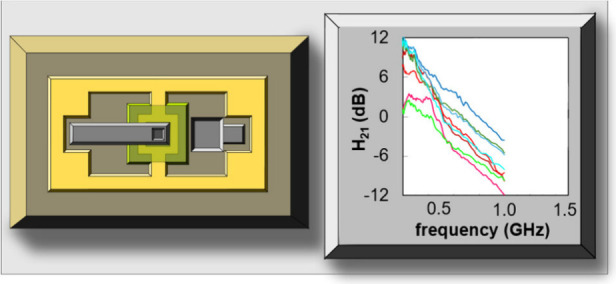

## 1 Introduction

The terahertz gap refers to the frequency range between microwave and infrared radiation (approximately 0.1–10 THz). It is technically difficult to access this spectral border area, in which conventional electronic and photonic components operate inefficiently. This makes the development of powerful sources, detectors and semiconductor devices for high-speed communication, imaging and spectroscopy a key technological challenge. While conventional high-speed transistors such as high-electron-mobility transistors, heterojunction bipolar transistors, or metal-oxide-semiconductor field-effect transistors are limited to cutoff frequencies below 1 THz,^1–3^ the graphene (Gr) based hot-electron transistor is potentially capable to operate up to 10 THz.^4^ This is due to the unmatched thin monolayer graphene base, which absolutely minimizes the base transit time of carriers. Such ultrashort base transit times are key for high-frequency HETs. A hot-electron transistor uses the transport of high-energy (“hot”) electrons across a thin potential barrier to enable faster and more efficient charge carrier injection compared to conventional devices. A voltage at the emitter-base barrier accelerates the electrons in the emitter to high energies (“hot electrons”). The electrons overcome the emitter-base barrier and move ballistically through the thin base. A Gr/semiconductor collector Schottky barrier allows only electrons with sufficiently high energy to pass into the collector. This study attempts to overcome the limitations of earlier GHETs, such as low output currents, low current gain, lack of output current saturation, narrow V_CB_ operating window, low on–off ratios, and limited scalability. Therefore, in this work we use n-type germanium (n-Ge) as the collector and Gr as the ultrathin base electrode. The E/B structure is varied in order to reduce the emitter-base barrier height qφ_BE_ and thickness. This could effectively reduce the turn-on voltage of the GHET and increase its output current density and current gain. Alternative E/B materials such as SiO_2_ or MoS_2_ with reduced interface contaminations and lower barrier heights qφ_BE_ are evaluated in the present work. The SiO_2_-based GHET achieves an exceptionally good common-base current gain α of 99.8%, which is already extremely close to the theoretical collection limit of 100%. Furthermore, the calculated common-emitter current gain β exceeds values of 400. We also present common-emitter Gummel plots for these devices, which are often omitted in other GHET publications.^5–8^ We determine record-high measured beta’s of around 42. For the MoS_2_ based device the turn-on voltage reduces and record high output current densities of ∼ 2000 A/cm^2^ are achieved.

Furthermore, ground-signal-ground (GSG) test structures are developed and the H_21_ current gain as a function of frequency is determined. Maximum cutoff frequencies (f_T_) of nearly 1 GHz are estimated for our lab scale SiO_2_ based devices with high current gain. To our knowledge, the present study is only the second publication in which measured RF characteristics of GHETs are shown. What makes our devices outstanding in this field is that they combine exceptional DC parameters (large on/off ratios, record-high and saturated output currents, a large V_CB_ operation window, very high current gains α and record-high β) with gigahertz operation. This is an important step toward the realization of ultrahigh cutoff frequency devices, for which significant device downscaling will be required in the future. However, the excellent data shown already in our lab-scale device demonstrate that the theoretical performance of the HET, up until ow only shown in simulations, are finally becoming also realistically achievable in real devices.

## 2 Experimental Section

### 2.1 Fabrication of the GHETs and DC/RF Characterization

The fabrication of the transistors in the conventional design is already described extensively in previous reports.^9–12^ The sophisticated GHET manufacturing process using GSG technology comprises, for example, more than 30 individual process steps in total and 6 lithography levels. In the first step, the n-type Germanium (1–3 Ohmcm, Umicore N.V./S.A.) wafers are cleaned using acetone, concentrated H_2_O_2_, Radio Corporation of America Clean, and hydrofluoric acid (HF) treatments. In the next step, 100 nm thick SiO_2_ is deposited by PECVD (ICP, 2000W, 3.5 mbar) using a gas mixture of silane and oxygen. Furthermore, lithography step 1 is applied to pattern the active device area by wet chemical etching in 5% HF solution. Moreover, lithography step 2 is used to fabricate the base and collector metal pads made from Ti/Ni (100 nm) by E-beam evaporation. In the next step, commercially available graphene on copper substrates is wet-transferred on top of the active device area.^13^ Unfortunately, Cu residues between n-Ge and graphene cannot be fully avoided following this approach.^14^ Subsequently, lithography step 3 is employed to pattern graphene by oxygen plasma (2 min). Then, 4 nm (±0.2 nm) thick SiO_2_ (1) is sputter deposited on top of graphene followed by lithography step 4 to locally deposit SiO_2_ (2) again by sputter deposition. The thickness of SiO_2_ (1) was measured on a separate p-type silicon substrate by ellipsometry using a standard SiO_2_ Cauchy-model. In addition, lithography step 5 is executed in order to dry-etch SiO_2_ (1) and SiO_2_ (2) by reactive ion etching (180 W, 17 sccm CHF_3_, 33 sccm Ar). Finally, lithography step 6, utilizing a double-resist scheme for better subsequent lift-off, is implemented to fabricate the emitter contact pad using again E-beam evaporation (Ti/Au – 220 nm). We fabricated 24 devices in total in conventional design (each for MoS_2_ and SiO_2_ based GHETs) and 40 devices in the GSG design. The results shown are mostly from champion devices.

#### 2.1.1 DC Characterization

A Keithley SCS 4200 semiconductor analyzer equipped with three independent source-measure units was used to record the IV characteristics. Micromanipulators fitted with 5-μm-radius tungsten probes were employed to contact the device’s emitter, base and collector pads, while the vacuum chuck provided backside contact to the wafer.

#### 2.1.2 RF Characterization

A Rohde & Schwarz ZVL6 vector network analyzer was used to measure the S-parameters of the embedded GHET devices as well as the open and short de-embedding structures. Subsequently, the S-parameters were postprocessed to de-embed the devices under test (DUTs). Finally, SPICE was used to determine the H-parameters of the DUTs in common emitter configuration and thus project the cutoff frequency f_T_ as shown in Figure 9.

### 2.2 CVD Growth of Large Area MoS_2_

Materials: Sodium molybdate (Na_2_MoO_4_ powder, 98.0%, Sigma-Aldrich), sulfur powder (99.98%, Sigma-Aldrich) were used without further purification.

Aqueous sodium molybdate solution preparation: For the molybdenum (Mo) source, 15 mM aqueous solution of sodium molybdate was prepared in ultrapure water (18.2 MΩcm, Membrapure).

Precursor substrate preparation: ∼ 1.5 cm × 1.5 cm Si substrate with a 300 nm thick SiO_2_ layer (Siltronix), root-mean-square (rms) roughness <0.2 nm) was used as growth substrate. To improve the hydrophilicity of the substrate as well as to increase the wettability of Mo precursor during spin coating, the substrates were exposed to O_2_ (10 standard cubic centimeters per minute (sccm)) plasma for 300 s. Further, the aqueous solution of sodium molybdate was spin-coated at 2000 rpm on a plasma-cleaned SiO_2_/Si substrate followed by heating at 65 °C for 5 min.

Controlled synthesis of large area MoS_2_ film: A two-zone furnace equipped with a quartz tube of 55 mm diameter (Carbolite Gero) was used for the growth in a similar manner as reported in.^15,16^ The growth substrate was heated rapidly (40 °C min^–1^) to 770 °C and kept constant for 15 min to grow large area MoS_2_ films.

### 2.3 Transfer of MoS_2_ Film on the HET Device

The as-grown monolayer MoS_2_ films (∼ 0.7 nm) were transferred onto the prefabricated HET devices via the wet transfer method described in.^17^ In brief, a layer of poly­(methyl methacrylate) (PMMA) was spin coated onto the as-grown MoS_2_. Thereafter, the sample was floated on top of potassium hydroxide aqueous solution to delaminate the PMMA/MoS_2_ film from the SiO_2_/Si substrate and subsequently washed with ultrapure water to remove residual KOH. Afterward, the PMMA/MoS_2_ film was placed onto the HET device and heated at 90 °C for 20 min to promote adhesion between MoS_2_ and the HET device. Next, the PMMA/MoS_2_/HET samples were immersed into acetone for 2 h to remove the PMMA, followed by isopropanol cleaning and nitrogen drying. At this stage, even though most of the PMMA is dissolved by acetone, some traces of PMMA can remain on the surface. To remove these residues and to further improve the adhesion between MoS_2_ and the HET devices, the samples were annealed at 150 °C for 3 h under inert atmosphere (Ar) followed by ultrasonic agitation (ultrasonic bath in acetone at a frequency between 40 and 80 kHz for 5 min at room temperature). The presence of monolayer MoS_2_ was verified via Raman measurements (see Figure S8).

## 3 Results and Discussion

### 3.1 DC Characterization of the SiO_2_-Based GHET in Conventional Design

The structure of the conventional GHET fabricated for the DC characterizations is shown in Figure 1 b). The core of the device is the SiO_2_ (MoS_2_)/ Gr/n-Ge junction. It is realized by exposing a 100 × 100 μm^2^ window in an Al_2_O_3_ insulation layer. Then, Gr is wet-transferred on top of the bare n-Ge and the base metallization. Furthermore, 4 nm thick SiO_2_ is sputter deposited or monolayer MoS_2_ is wet transferred on top of graphene. The aim of using these two emitter-base materials was to decrease the E/B barrier height qφ_BE_. This barrier is 4 eV for previous hBN/Gr devices,^9^ and reduces to 3.6 eV with SiO_2_/Gr^18^ and 0.6 eV with MoS_2_/Gr.^19^ With previous devices using multilayer hexagonal boron nitride (hBN, 4 nm), a moderate common-base current gain α of about 87% was achieved. The highest observed output current of this GHET was 800 A/cm^2^.^9^ Thereby, hBN was transferred on top of Gr based on a copper wet-etch process, which leads to Cu residues at the hBN/Gr interface. These copper residues act as scattering sites, reducing the current gain and output current density.^20^ The advantage of the sputter-deposited SiO_2_ is that the interface between SiO_2_ and Gr is probably free of contaminations reducing the number of scattering sites. For wet transferred MoS_2_ potassium residues might contaminate the interface to Gr, potentially reducing the current gain of these devices.

**Figure 1:**
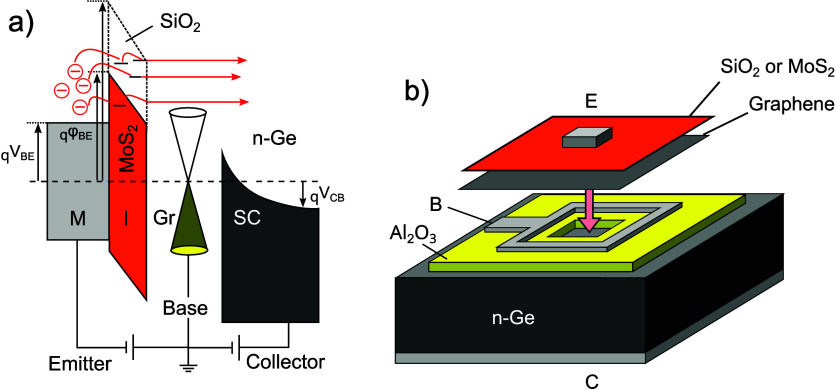
a) Simplified band scheme of the metal (M), insulator (I), graphene (Gr), semiconductor (SC) hot electron transistor in the on-state. b) Three-dimensional representation of the hot electron transistor with a SiO_2_ or MoS_2_ emitter-base (E/B) insulator, a monolayer graphene base (B), and an n-germanium collector (C) semiconductor (conventional design for DC characterization).

Moreover, reducing the barrier heights of the SiO_2_ and MoS_2_ based devices could favor Fowler-Nordheim tunnelling at decreased emitter-base voltages (turn-on voltage). However, contrary to expectations, the measured base-emitter currents of our devices could not be explained by either the Fowler-Nordheim tunneling process or the direct tunneling process. Instead, these BE-currents obey to the Nearest Neighbor Hopping (NNH) model (see Figure S1 and Figure S2). Thus, the intended reduction of the base-emitter barrier height therefore plays a subordinate role, as it is not taken into account in the NNH model. In MoS_2_ devices with low barrier, carriers could also overcome the barrier through thermionic emission at high bias voltages. Figure 1 a) illustrates the simplified band scheme of the GHET in the on-state that reflects the situation described above. The common-base output characteristics of the SiO_2_ based GHET are depicted in Figure 2 a). The collector current highly scales with the emitter voltage. Thus, on–off ratios larger than 10^3^ are achieved. Thereby, the collector current saturates for increased collector-base voltages, which is important for the high-frequency operation. A slight negative differential resistance (NDR) is visible in the output curves at higher emitter voltages. Explicitly, for a certain V_CB_ the collector current drops from a higher current to a lower current, yielding maximum peak to valley ratios of about 1.2. A stable NDR behavior could possibly be exploited for high-frequency oscillators.

**Figure 2:**
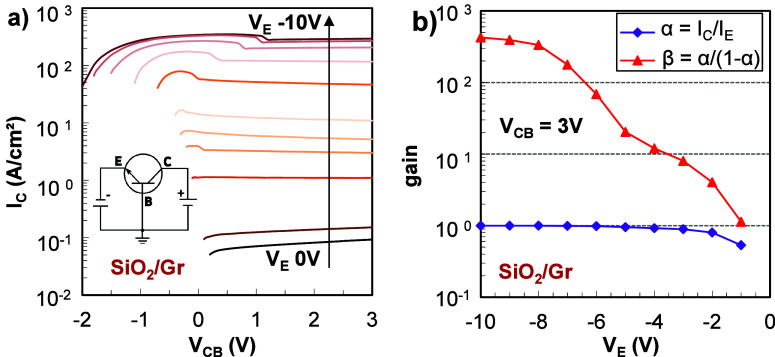
a) Common-base output characteristics of the SiO_2_/ Gr/n-Ge hot electron transistor with the emitter voltage as a parameter. b) Measured common-base gain α and calculated common-emitter gain β of the SiO_2_/Gr based hot electron transistor as a function of the emitter-voltage.

The measured common-base gain α and calculated common-emitter gain β of the SiO_2_-based GHET are shown in Figure 2 b). At increased emitter voltages, α is as high as 99.8% and β is up to 425. When calculating β, the leakage current I_C_ (V_E_ = 0 V) was not subtracted because the leakage currents are very low and only have a minor influence on the input-driven collector currents. The observed gain is comparable to standard silicon NPN bipolar transistors and is strongly increased compared to previous devices with an hBN/Gr E/B structure (α_hBN_ = 87%, β_hBN_ = 7).^9^ The increased current gain of the SiO_2_-based GHET is attributed to reduced interface contaminations at the E/B interface.

The common-emitter output characteristics of an average SiO_2_-based GHET device with I_B_ as a parameter are shown in Figure 3 a). A distinctive amplification with I_C_ larger than I_B_ for V_CE_ > 5 V can be observed. Figure 3 b) presents the common-emitter Gummel plot of the SiO_2_-based champion device showing I_C_ and I_B_ as a function of the base voltage. Thereby, the collector current I_C_ is larger than I_B_ in the entire V_B_-range. This results in measured common-emitter current gains β = I_C_/I_B_ of larger than one. This confirms that β > 1 is not only achieved by calculation from measured common-base gains α but is also verified by direct common-emitter measurements. Such common-emitter Gummel plots, standard for classical silicon bipolar junction transistors, are mostly omitted in other GHET publications.^5–8^ The maximum measured common-emitter current gain β = I_C_/I_B_ (ΔI_C_/ΔI_B_) of the SiO_2_ based GHET examined in this study is about 20 (42) (see Figure 3 b), which is lower than calculated from common-base characteristics. The reason is that I_C_ is not exceeding values of 10^2^ A/cm^2^ in the Gummel configuration. In comparison, I_C_ saturates at values slightly above 10^2^ A/cm^2^ for V_E_ = −7 to −10 V according to the common-base output characteristics (see Figure 2 a). The higher common-base output current could be related to a thermal runaway. It should be noted that a monolayer of graphene will likely heat up by at least 30 to 40 K when exposed to a current of larger than 1 mA. (the 1 mA corresponds to a current density above 10^1^ A/cm^2^ for our devices). The elevated temperature also increases the reverse saturation current density of the Gr/n-Ge diode (see Figure S4) reflecting in a positive offset current in the common-base output characteristics for I_C_ > 10^1^ A/cm^2^. However, when measuring the Gummel plot in common-emitter mode, the measurement time is strongly reduced, leading to lower parasitic heat-up. Furthermore, the maximum transconductance g_m_ of the best SiO_2_-based GHET is approximately 1 mS.

**Figure 3:**
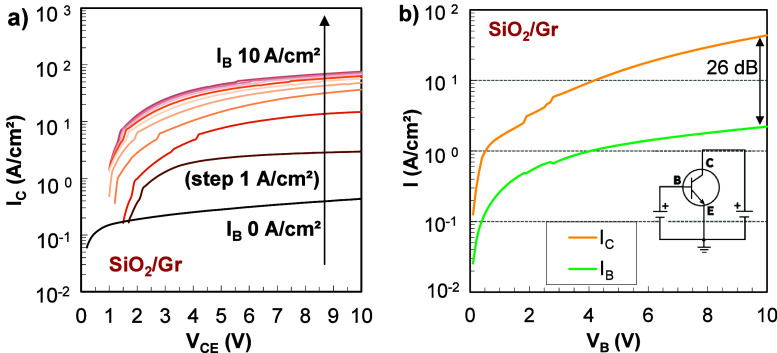
a) Common-emitter output characteristics of an average SiO_2_/ Gr/n-Ge GHET with I_B_ as a parameter. b) Gummel plot of the best SiO_2_/ Gr/n-Ge hot electron transistor showing I_C_ and I_B_ as a function of the base voltage. Maximum measured current gain β = I_C_/I_B_ = 20 (26 dB).

### 3.2 DC Characterization of the MoS_2_-Based GHET in Conventional Design

Moreover, GHETs with further reduced E/B barrier heights qφ_BE_ and thicknesses were fabricated. To this end, monolayer MoS_2_ was transferred on top of graphene by means of a wet transfer approach. Compared to GHETs with hBN transferred onto Gr, MoS_2_ transfer does not require a copper substrate, as MoS_2_ is grown on SiO_2_ via chemical vapor deposition (CVD) (see experimental section for details). To release MoS_2_ from the SiO_2_ substrate potassium hydroxide is used.

In Figure 4 a) the common-base output characteristics of the MoS_2_ based GHET are presented. Compared to the SiO_2_-based GHET, the collector current increases more rapidly at lower emitter voltages. Thus, the turn-on voltage is decreased, which can be explained by the lower thickness of MoS_2_.

**Figure 4:**
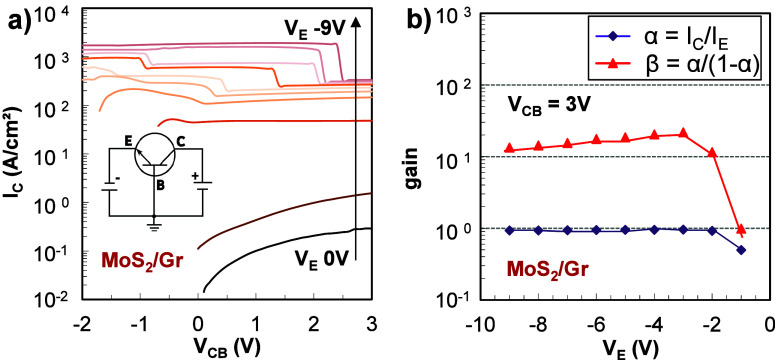
a) Common-base output characteristics of the MoS_2_/ Gr/n-Ge hot electron transistor with the emitter voltage as a parameter. b) Gain of the MoS_2_/Gr based hot electron transistor as a function of the emitter-voltage.

In addition, the NDR effect is more pronounced for the MoS_2_ based GHET. Peak-to-valley current ratios of about 6 are achieved. While the valley collector currents are almost the same at increased emitter voltages for both the MoS_2_ and SiO_2_ based GHETs, the peak collector currents of the MoS_2_ based GHET are substantially increased. This results in record collector currents of around 2000 A/cm^2^ for the MoS_2_ based GHET. The current gain of the MoS_2_ based GHET as a function of the emitter voltage is shown in Figure 4 b). The maximum measured common-base current gain α is 96% and the calculated β reaches maximum values of about 20. The current gain is stable across a wide range of emitter voltages. During the measurement of the device under a constant I_E_ condition (see Figure S5), the maximum current gain α decreases slightly. Compared to the SiO_2_-based GHET, the current gain of the MoS_2_-based GHET is reduced. Eventually, potassium residues at the MoS_2_/Gr interface, which may result from the MoS_2_ wet transfer, are responsible for this behavior. These contaminants may act as scattering sites, repelling part of the incoming high-energy emitter electrons.

Furthermore, the transfer curves for the MoS_2_/Gr and SiO_2_/Gr GHETs are compared in Figure 5. As can be seen, the slope of I_C_ vs V_E_ is increased for the MoS_2_/Gr based GHET. The reason for that is the reduced thickness of the MoS_2_ monolayer. This results in lower turn-on voltages for the MoS_2_/Gr based GHET. At higher emitter voltages, the collector currents for both devices are limited by the series resistance between collector and base and space charge limited conduction (SCLC) in the collector rather than by the E/B junction. The main parameter which determines SCLC is the collector thickness. In this study, we used 500 μm thick n-type germanium. Future devices with strongly decreased semiconductor thicknesses and optimized series resistances could enable much higher output currents beyond 10^4^ A/cm^2^. Next, GHETs with front contacts for both the emitter, base and collector terminals are developed for high-frequency test environments.

**Figure 5:**
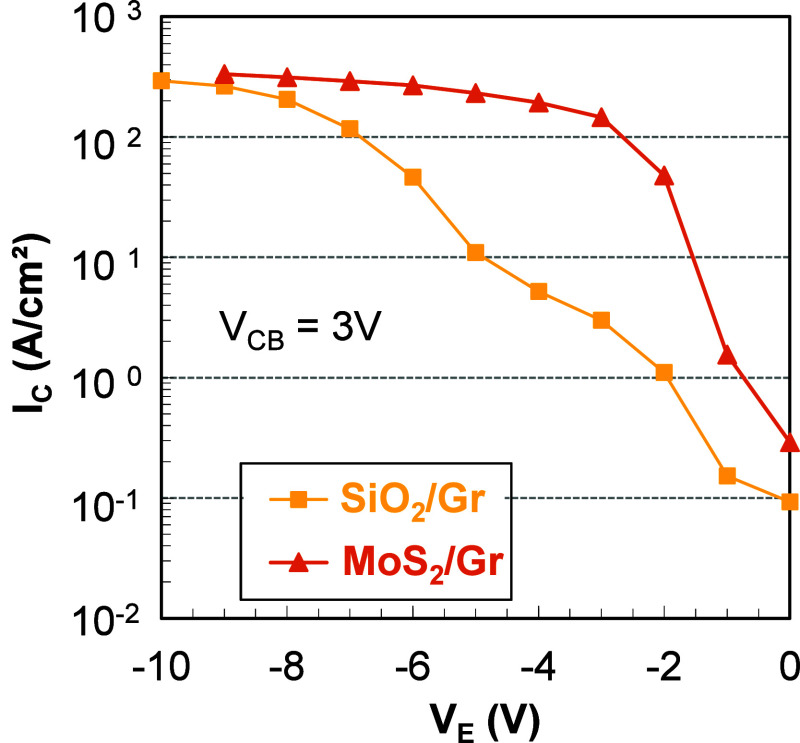
Collector current as a function of the emitter voltage for SiO_2_/Gr and MoS_2_/Gr-based devices.

### 3.3 High-Frequency Characterization of the SiO_2_-Based GHET

We selected the SiO_2_-based GHET for further investigations on its high-frequency performance due to its most promising DC characteristics. Therefore, a ground-signal-ground test structure with all contact pads at the front side of the device was designed. Figure 6 shows a schematic cross section of the new test structure and Figure 7 illustrates a top view image of a fabricated device. Thereby, in the top part of Figure 7 the manufactured device is displayed, while the middle and bottom part of Figure 7 show the open and short calibration structures used for de-embedding of the RF-measurements.

**Figure 6:**
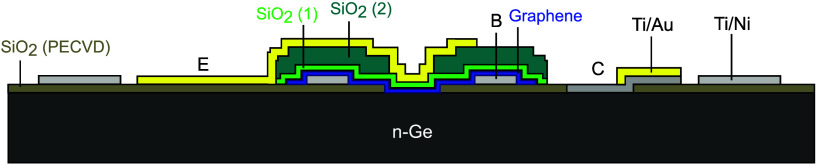
Cross section of the SiO_2_/Gr based GHET in the ground-signal-ground (GSG) configuration used for its high-frequency characterizations.

**Figure 7:**
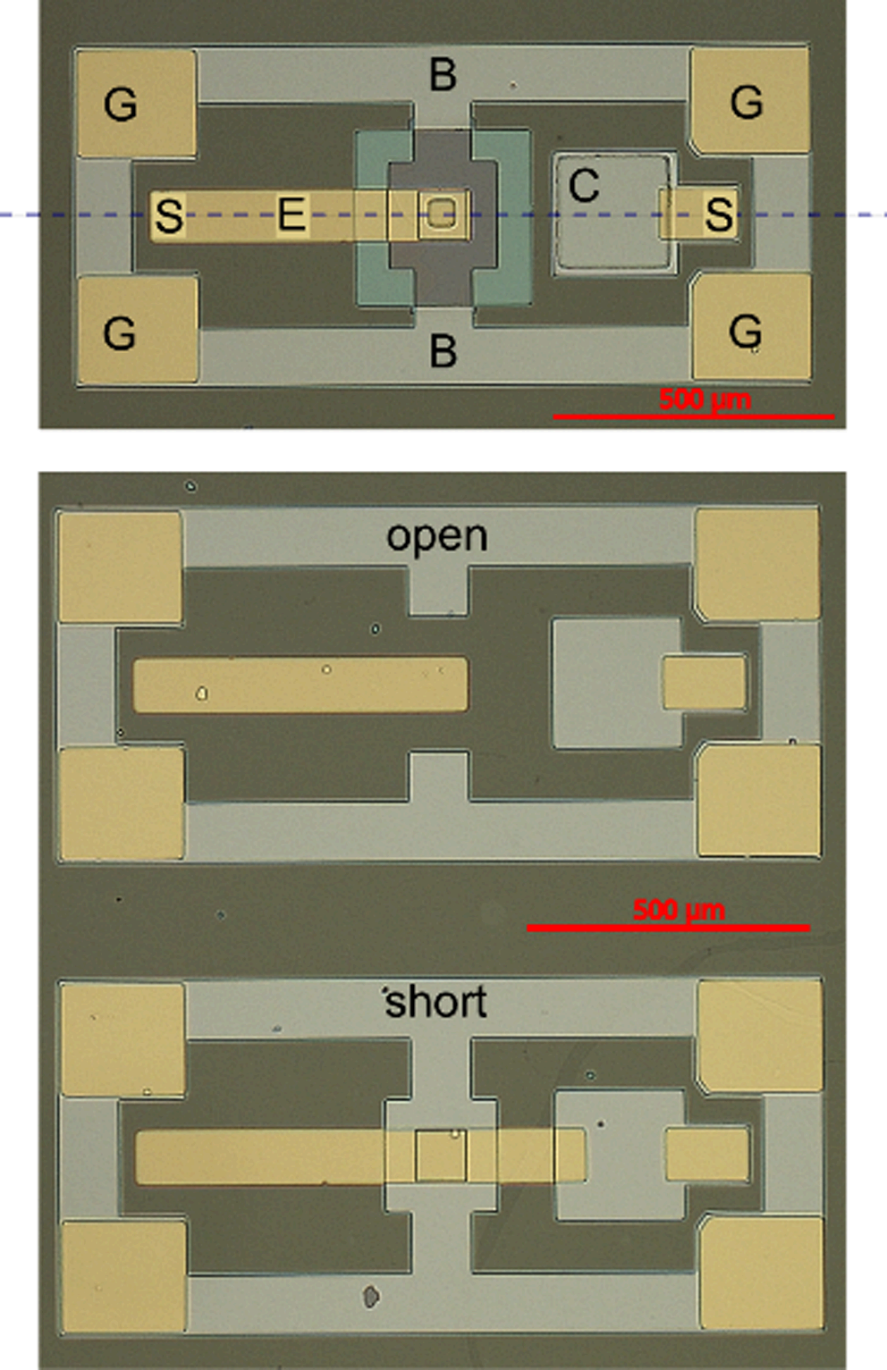
Top view microscope image of a fabricated SiO_2_/Gr based hot electron transistor in the ground-signal-ground (GSG) configuration (top). Open (middle) and short (bottom) calibration structures used for de-embedding.

Furthermore, the dashed line in Figure 7 above indicates the point where the cross-section in Figure 6 was drawn. From this cross section it can be deduced that three different SiO_2_ layers are applied. SiO_2_ (PECVD) is used to fully insulate the base metallization and partly insulate the collector and emitter metallization from n-Ge. Moreover, SiO_2_ (1) serves as the 4 nm thick E/B insulator, while the 60 nm thick SiO_2_ (2) is used to insulate the emitter metallization from graphene. The latter is necessary because otherwise a short between Gr and emitter to the left of the active Gr/n-Ge junction area will lead to the failure of the devices. This is because a 4 nm thick layer of sputter deposited SiO_2_ (1) is not fully insulating the emitter metallization from the graphene. The active area of the GHETs with Gr in direct contact with n-Ge was varied from 25 × 25 μm to 50 × 50 μm and 100 × 100 μm.

Figure 8a) shows the common-base output characteristics of a 50 × 50 μm large device in the adjusted ground-signal-ground technology. It is obvious that the maximum output current is lower compared to the SiO_2_ based devices fabricated conventionally (see Figure 2a). Thus, the on–off ratio of the GSG device is also lower with values of about 1.5 × 10^1^ (V_CB_ = 1 V). The output current saturation is maintained even with the GSG devices. In particular, GSG-devices with different sizes (25 × 25 μm, 100 × 100 μm) exhibit similar output characteristics as the 50 × 50 μm device.

**Figure 8:**
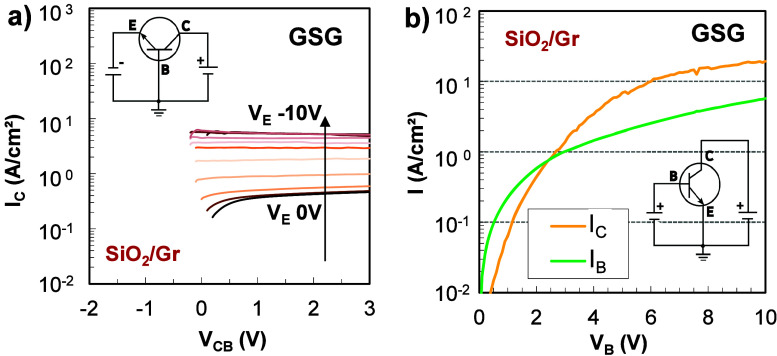
a) Common-base output characteristics of a 50 × 50 μm large SiO_2_/ Gr/n-Ge GHET in the ground-signal-ground (GSG) contact configuration with the emitter voltage as a parameter. b) Gummel plot of a 50 × 50 μm large SiO_2_/ Gr/n-Ge GHET in the ground-signal-ground (GSG) contact configuration showing I_C_ and I_B_ as a function of the base voltage.

So far, the reason for the lower output currents of the GSG devices is not fully understood. It must be noted that during the GSG test structure fabrication two additional lithography steps are included after graphene transfer, which might lead to additional photoresist residues between SiO_2_ and the emitter contact. Another difference between conventional device fabrication and the GSG device is related to the applied n-Ge insulator. In case of conventional fabrication, 60 nm thick Al_2_O_3_ is used to insulate the base metal pad from the n-Ge. On the other hand, for the GSG design 100 nm thick SiO_2_ (PECVD) is applied for the same purpose. The idea behind was to establish a thicker and more robust etch stop material for RIE of SiO_2_ (1) and SiO_2_ (2), which represents an additional process step in GSG manufacturing. In any case, the different applied insulators could influence the Gr/n-Ge interface, because the active device area must be exposed from Al_2_O_3_/SiO_2_ by wet chemical etching in hydrofluoric acid solution. We also checked whether the smaller n-Ge/collector front contact in the GSG design could be the reason for the lower output currents. Therefore, additional measurements utilizing the metallized backside of n-Ge as the new collector of the GSG devices were implemented. However, the output currents were also lower for this reference measurement eliminating the front-contact as origin of the reduced I_C_ values. In addition, IV-measurements verified that the metal/n-Ge collector front contact resistance can be neglected (see Figure S3). Finally, for the GSG production, n-Ge wafers from a new batch with almost identical properties were used, as the previous wafer batch was no longer available. This might also affect the currents of the GSG devices.

Moreover, the Gummel plot of the GHET in GSG technology is shown in Figure 8 b). The measured common-emitter current gain β, although slightly lower than for conventionally fabricated devices (see Figure 3 b), remains above one for V_B_ > 2 V. The maximum measured common-emitter gain β = I_C_/I_B_ is around 4 (12 dB). This result is a good starting point for initial RF measurements up to the GHz range. To de-embed the GHET, i.e., to eliminate the extraneous effects arising from the electrode–substrate interfaces, and thus revealing the GHETs intrinsic RF characteristics, the established open-short de-embedding has been employed.^21^ The on-chip open and short de-embedding structures used for this are shown in the bottom part of Figure 7. Figure 9 illustrates the de-embedded current gain H_21_ as a function of frequency. A decrease of H_21_ with increasing frequency can be generally observed (gain roll-off −24 dB/dec., Figure S7). No significant influence of the investigated active device area (red curves: 100 × 100 μm large devices, blue curves: 50 × 50 μm large devices, green curves: 25 × 25 μm large devices) could be determined. Lateral downscaling mainly affects the delay time specified by R_E_C_BC_, an important limiter of f_T_.^22^ However, in this study, the scaling effects could be masked by parasitic effects (e.g., extrinsic C_BC_, substrate loss effects). In addition, the device-to-device variability (see Table 1) still needs to be improved to identify true scaling effects. The cutoff frequency f_T_ at which the common-emitter current gain drops to 1 (H_21_ = 0 dB) can easily be extracted from Figure 9. Thus, the maximum measured cutoff frequency f_T_ max is 0.8 GHz for a 50 × 50 μm large device.

**Figure 9:**
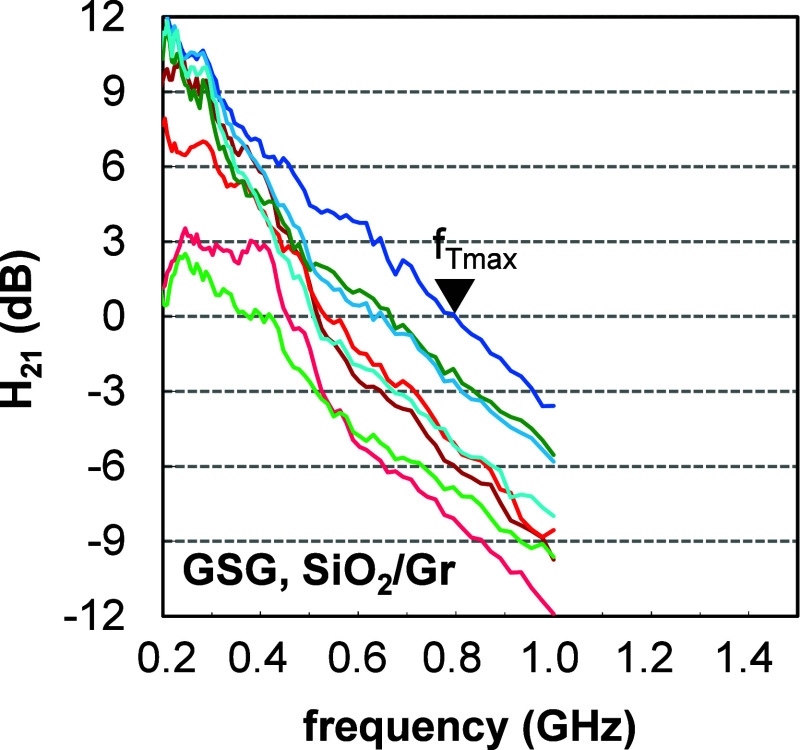
RF measurements of the SiO_2_ based GHET with ground-signal-ground (GSG) front side contact pads. Current gain H_21_ as a function of frequency: at a fixed V_CB_ of 2 V and V_E_ = −10 V. The maximum measured cutoff frequency f_T_ max = 0.8 GHz. Red curves: 100 × 100 μm large devices, blue curves: 50 × 50 μm large devices, green curves: 25 × 25 μm large devices.

**Table 1:** Average, Maximum, and Minimum Values of Selected Device Parameters (α_max_, β_meas_, I_Cmax_, f_T_) for Numerous Measured GHET Devices with a SiO_2_/MoS_2_ Emitter-Base Composition in Conventional Design (Lines 1-6) and in GSG Design (Lines 7-9)

		α_max_ (%)	β_meas_ (I_C_/I_B_)	I_Cmax_ (Acm^–2^)	f_T_ (GHz)
**GHET SiO**_**2**_	avg.	97.65	10.68	141	
	max	99.77	19.72	327	
	min	89.97	1.46	8	
**GHET MoS_2_**	avg.	93.10	1.29	947.5	
	max	95.82	1.63	1960	
	min	91.86	0.62	520	
**GHET SiO**_**2**_ **GSG**	avg.	43.88	1.51	1.51	0.58
	max	69.63	3.85	3.92	0.80
	min	21.33	0.15	0.25	0.42

Furthermore, Table 1 shows average, maximum, and minimum values of selected device parameters for numerous measured GHET devices. The values suggest that the parameter deviations for SiO_2_-based devices, especially in the GSG design, are greater than for MoS_2_ devices. In the future, improved fabrication methods will be required to increase the reproducibility and robustness of the manufacturing process.

### 3.4 Benchmarking with State of the Art

In 2017, a GHET with a GaN/AlN/Gr/WSe_2_ structure was reported.^23^ For the first time, a high current density (50 A/cm^2^) and a high current gain (α_max_ = 75%) were combined. For early devices with a MoS_2_ base material α = 95% was achieved and a cutoff frequencies of 7.6 MHz was claimed based on calculations of the E/B charging time and C/B delay time.^24^ A silicon based GHET with estimated f_T_ = 26 MHz was reported in.^10^ However, the current gain of this device was still too low (α = 2%). A further improved hBN/Gr/n-Ge based GHET with α of 87% and a record output current of 800 A/cm^2^ was presented in 2024.^9^ Furthermore, two promising GHETs with high current gains α of 99% and output current densities above 400 A/cm^2^ were introduced in 2019.^7,8^ The first device consists of a n-silicon/Gr/n-germanium structure but the on–off ratio and output current saturation were not satisfiable. A cutoff frequency of 1.2 GHz was predicted for this device based on calculations of the emitter charging time. The second device utilizes a Gr/hBN/Gr/WSe_2_ composition with all 2D layers stacked on top of each other by exfoliation and transfer techniques. Therefore, the scalability of this approach may currently be difficult. In addition, the device can be operated only in a very small V_CB_ window. The first GHET for which the H_21_ current gain was actually measured as a function of frequency was released in 2021.^5^ A cutoff frequency of 65 GHz was demonstrated. Despite these outstanding results, the moderate common-emitter current gain β of 2.7 leaves room for improvement and also the output current saturation and on–off ratio of this device still remained to be issues.

In summary, the results presented in this work are at the forefront of the current state of the art and in some cases even exceed it (output current ∼ 2000 A/cm^2^, measured common-emitter gain β ∼ 42). Problems of previous devices such as limited scalability, lack of output current saturation, or low on–off ratios are solved for our devices. The promising results of our germanium based technology can also be transferred to silicon substrates in the future. This could, for the first time, enable the development of cost-effective, scalable and reliable RF amplifiers with ultrahigh cutoff frequencies. Table 2 summarizes the most important device parameters described in the literature in comparison to the results obtained in this work.

**Table 2:** Most Important Device Parameters of Selected HETs Reported in the Literature^a^

						J_c_	f_T_	
Year	EB	base	BC	β_meas_	α_max_	(Acm^–2^)	(GHz)	ref.
2013	SiO_2_	Gr	Al_2_O_3_	-	0.065	2 × 10^–5^	-	^18^
2015	SiO_2_	MoS_2_	HfO_2_	4	0.95	10^–4^	-	^24^
2017	AlN	Gr	WSe_2_	4–6	0.75	50	-	^23^
2015	TmSiO/TiO_2_	Gr	Si	0.4	0.28	4	-	^25^
2024	hBN	Gr	n-Ge	-	0.87	800	-	^9^
2019	n-Si	Gr	n-Ge	-	0.99	692	-	^7^
2019	hBN	Gr	WSe_2_	-	0.999	400	-	^8^
2021	SiO_2_	Gr	WSe_2_	2.7	0.992	200	65	^5^
this work	SiO_2_	Gr	n-Ge	42	0.998	327	0.8	
this work	MoS_2_	Gr	n-Ge	1.6	0.96	1960	-	

a EB – emitter base barrier material, CB -collector base barrier material.

## 4 Conclusions

To conclude, a novel graphene-based hot-electron transistor technology platform is introduced. This transistor technology potentially allows for ultrahigh cutoff frequencies beyond 1 THz. An innovative material combination of SiO_2_/graphene/n-germanium or monolayer MoS_2_/graphene/n-germanium was evaluated. The effect of different emitter-base compositions was investigated. It could be observed that the SiO_2_/Gr based hot-electron transistor performed best in terms of the measured current gain α (99.8%) and calculated current gain β (425). Furthermore, an excellent measured common-emitter current gain β of 42 was achieved. However, the reason behind is rather related to a reduced number of scattering sites instead of a reduced barrier height. A further reduction of barrier thickness was achieved with the MoS_2_/Gr based hot-electron transistor. The current gain for this device was slightly lower compared to the SiO_2_/Gr device, but the output current reached record values of 2000 A/cm^2^. The output currents as a function of collector-base voltage are saturating and the devices operate in a wide V_CB_ window, overcoming the limitations of previously reported hot-electron transistor devices. In addition, the turn-on voltage of the MoS_2_-based device could be decreased to 2 V compared to 7 V for the SiO_2_/Gr based device. Furthermore, the presented devices utilize scalable fabrication methods. The experimentally achieved cutoff frequency of the SiO_2_-based GHET is close to 1 GHz. In the future, further optimizations regarding collector thickness and collector-base series resistance could enable devices with output currents above 10^4^ A/cm^2^, a prerequisite for THz operation. These outstanding results are an important step toward functional graphene based hot-electron transistors operating at ultrahigh speed.

## Supplementary Material



## Notes

Characterization of the BE-junctions; Metal/n-germanium Schottky contact; Base-collector two-terminal characterization; Additional three-terminal characterizations; H21 roll-off; Raman measurement of monolayer MoS_2_ (PDF)

## Abbreviations

GHET: graphene hot-electron transistor
E: emitter
B: base
C: collector
Gr: graphene
DC: direct current
GSG: ground-signal-ground
RF: radio frequency
NDR: negative differential resistance
CVD: chemical vapor deposition
PECVD: plasma-enhanced chemical vapor deposition
SCLC: space charge limited conduction
f_T_: cutoff frequency
2D: two-dimensional
HF: hydrofluoric acid
ICP: inductively coupled plasma
DUT: device under test
SPICE: Simulation Program with Integrated Circuit Emphasis
