# Supplementary material for: Gigahertz Cutoff Frequencies and High Gain in Graphene-Based Hot-Electron Transistor Enabled by Material Engineering
Source: ACS Appl Mater Interfaces. 2026 Jun 20;18(25):35812–20. doi: 10.1021/acsami.6c04685 (PMC13339028; doi:10.1021/acsami.6c04685)
Supplement: Supplementary file 1 [file am6c04685_si_001.pdf]

SUPPORTING INFORMATION

Gigahertz Cutoff Frequencies and High Gain in Graphene Based Hot-Electron Transistor Enabled by Material Engineering

Carsten Strobel[1,], André Heinzig[1], Andre Hiess[1], Martin Knaut[1], Md Tarik Hossain[2], Andrey Turchanin[2], Tilo Meister[3], Frank Ellinger[3], Jens Trommer[4], Viktor Havel[4], Thomas Mikolajick[1]*

1 Institute of Semiconductors and Microsystems, Chair of Nanoelectronics, Technische Universität Dresden, Nöthnitzer Straße 64, 01187 Dresden, Germany

2 Friedrich-Schiller-Universität Jena, Lessingstraße 10, 07743 Jena, Germany

3 Chair of Circuit Design and Network Theory, Technische Universität Dresden, Helmholtzstraße 18, 01069 Dresden, Germany

4 NaMLab gGmbH, Nöthnitzer Str. 64 a, 01187 Dresden, Germany

*carsten.strobel@tu-dresden.de

CHARACTERIZATION OF THE BE-JUNCTIONS

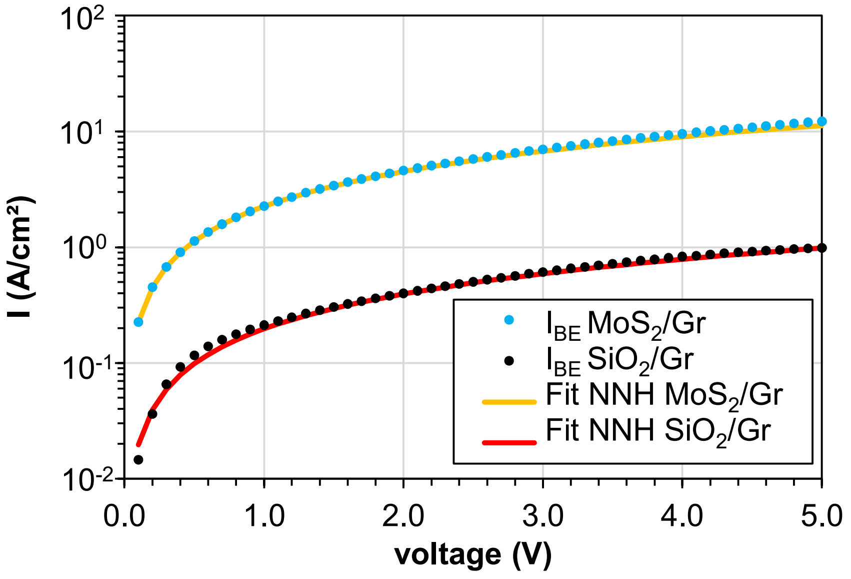

Figure S 1: Measured two-terminal base-emitter currents of the SiO_2/Gr and MoS_2/Gr junctions (dots) and fitted currents by the nearest-neighbor-hopping model (NNH, solid lines).

Figure S 1 shows the measured and fitted two-terminal currents of the individual base-emitter junctions. Thereby, the base-emitter current of the MoS_2/Gr junction is about one order of magnitude larger than the base-emitter current of the SiO_2/Gr junction. Against expectations, the currents can best be described using the Nearest Neighbor Hopping (NNH) method [1]:

$$J_{NNH} = \sigma_0 \exp\left(\frac{-T_0}{T}\right) \cdot E \qquad (S1)$$

where σ_0 is the electrical conductivity at temperature T_0. Neither Fowler-Nordheim Tunneling nor direct tunneling describe the measured currents very well. Thus, it can be concluded that the intended base-emitter barrier height reduction plays a subordinated role as it is not reflected in the NNH model.

Furthermore, the temperature dependence of J_{NNH} was investigated (see Figure S 2). A linear fit of $\ln(J_{NNH}/E)$ vs. 1/T verifies the assumption of the NNH-model with an activation energy of the hopping process of 0.154 eV for SiO_2 and 0.082 eV for MoS_2.

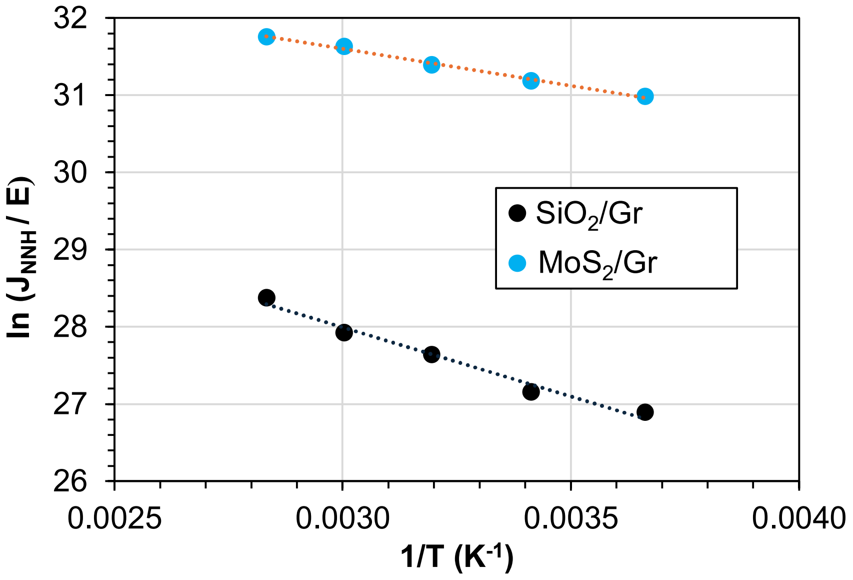

Figure S 2: Arrhenius plot of $\ln(J_{NNH}/E)$ vs. 1/T for the MoS_2/Gr (blue circles) and SiO_2/Gr (black circles) junctions.

METAL/N-GERMANIUM SCHOTTKY CONTACT

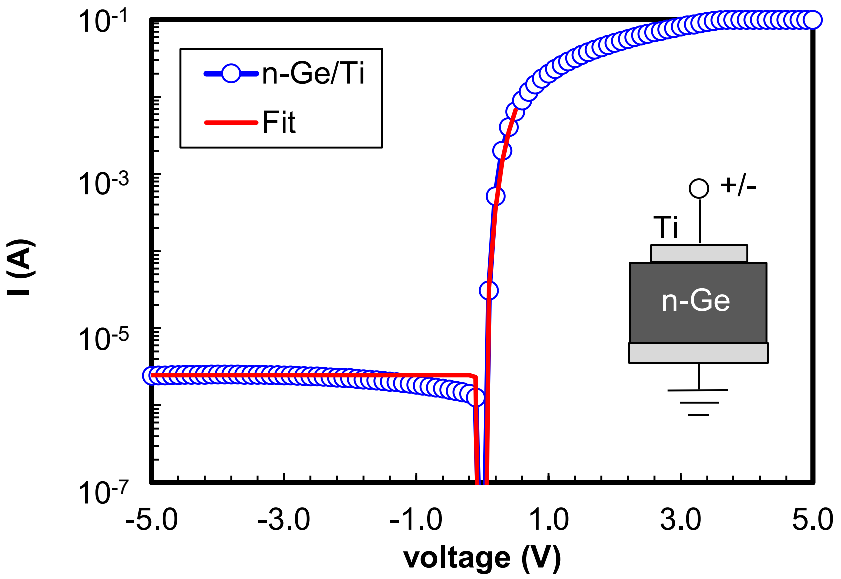

Figure S 3: Measured IV-curve (blue dots) and fit of the Ti/n-Ge Schottky junction (red solid line).

The metal/n-Ge front contact is further investigated because a large contact resistance could impact the RF performance of the GHET through impedance losses. This metal/n-Ge junction represents the collector contact at the front side of the GSG device. Figure S 3 shows the measured IV-curve of a 200x200 µm² large Ti/n-Ge junction (blue dots). A clear rectification can be observed at this Ti/n-Ge top Schottky contact. The full-area metallized back side of n-Ge can be assumed to be an ohmic contact due to the very large junction area. Thus, the observed rectification can be assigned to the confined metal/n-Ge top contact. The fit of the Ti/n-Ge junction current is obtained according to:

$$I = I_0 \left[exp\left(\frac{q(V - IR_s)}{nkT} \right) - 1 \right] \qquad (S2)$$

with I_0 the reverse saturation current, R_s the series resistance, and n the diode ideality factor. The extracted series resistance R_s of this Schottky diode is 32 ohms, most of which is due to the n-Ge bulk resistance. Thus, the contact resistance of the Ti/n-Ge junction is assumed to be negligible and will not strongly impact the RF performance of the GHET. It must be noted, that the Ti/n-Ge collector junction of the GSG-GHET is biased in forward direction during the device operation. This means that the reverse blocking of the Ti/n-Ge Schottky junction does not play a significant role in the operation of the device.

BASE-COLLECTOR TWO-TERMINAL CHARACTERIZATION

Figure S 4 illustrates IV-curves of the Gr/n-Ge base-collector junction of devices fabricated in conventional design.

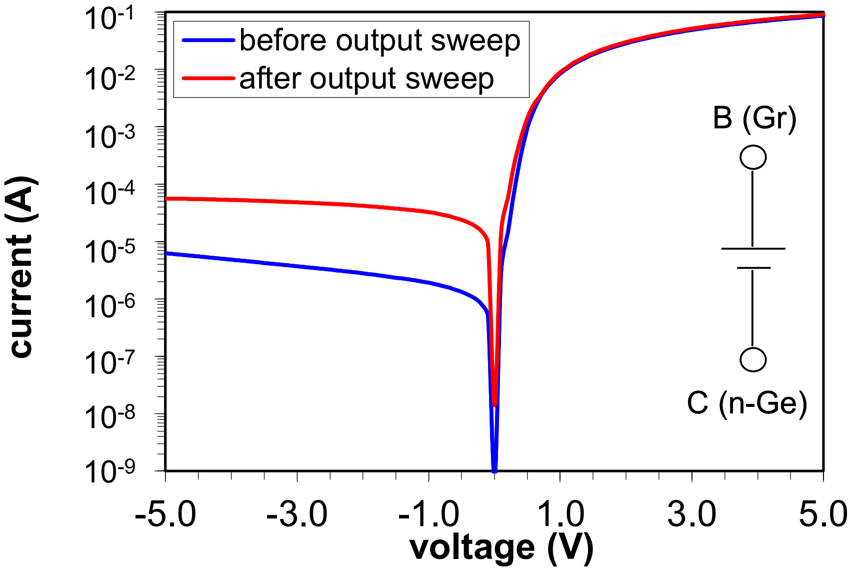

Figure S 4: Two-terminal IV-curves of the base-collector (BC) junction of devices fabricated in conventional design before and after measuring the three-terminal output characteristics I_C-V_{CB} (V_E = 0…-10V) of the same device.

The blue curve represents the initial state, while the red curve represents the state after measuring the complete three-terminal output characteristics of the same device. It can be observed that the reverse current of the Gr/n-Ge Schottky diode increases about one order of magnitude compared to the as-deposited state. This is attributed to current-induced heating of the Gr/n-Ge junction during the three-terminal characterization process.

ADDITIONAL THREE-TERMINAL CHARACTERIZATIONS

In Figure S 5 the common-base output characteristics of a MoS_2-based device in conventional design with I_E as a parameter are shown. The collector leakage current for I_E = 0 A/cm² is low and comparable to the leakage current for measurements with V_E = 0 V. Specifically, no additional leakage current by a V_{CB}-induced modulation of the BE tunneling barrier can be observed.

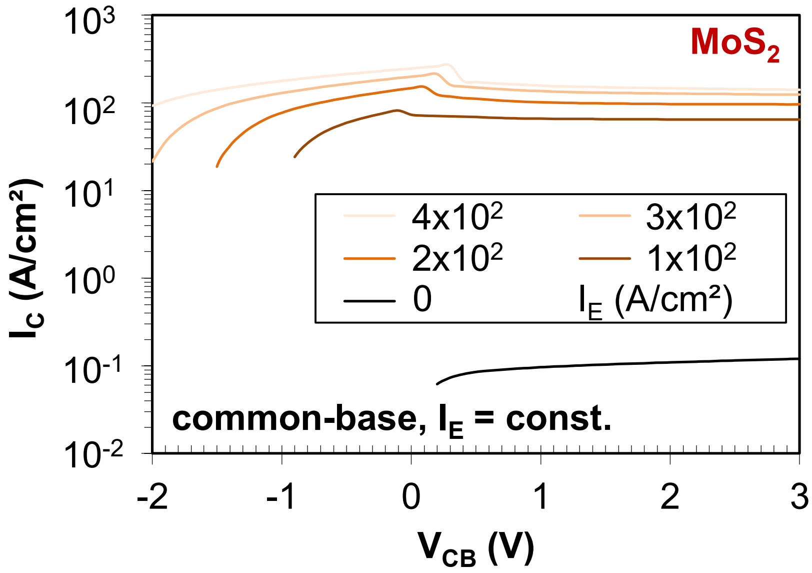

Figure S 5: Common-base output characteristics of a MoS_2/Gr based device in conventional design with I_E as a parameter.

Especially at high input levels (emitter currents, emitter voltages), the leakage current is orders of magnitude lower than the input driven collector currents. Therefore, to calculate β from α, in a

first approximation no subtraction of the leakage current I_C (I_E = 0 A/cm², V_E = 0V) from the input driven collector currents is required.

Figure S 6 shows the emitter current as a function of the collector-base voltage with the emitter voltage as a parameter. Thereby, an SiO_2-based device in conventional design was selected. It can be observed, that V_{CB} is not much affecting the emitter current. Thus, it can be assumed that the collector current modulation as observed in Figure 2 a) of the manuscript truly originates from hot-electron transport rather than bias-induced changes in emitter injection.

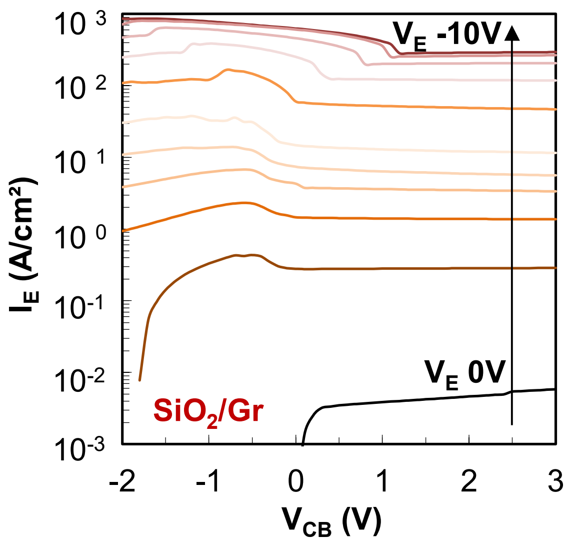

Figure S 6: Emitter current as a function of the collector-base voltage with the emitter voltage as a parameter (SiO_2-based device in conventional design).

H21 ROLL-OFF

Figure S 7 depicts the H_{21} current gain of the SiO_2 based GHET in the GSG design as a function of the frequency. Thereby, the frequency axis is presented in the logarithmic scale. From this plot, the gain roll-off slope can be better analyzed, yielding -24 dB/dec.

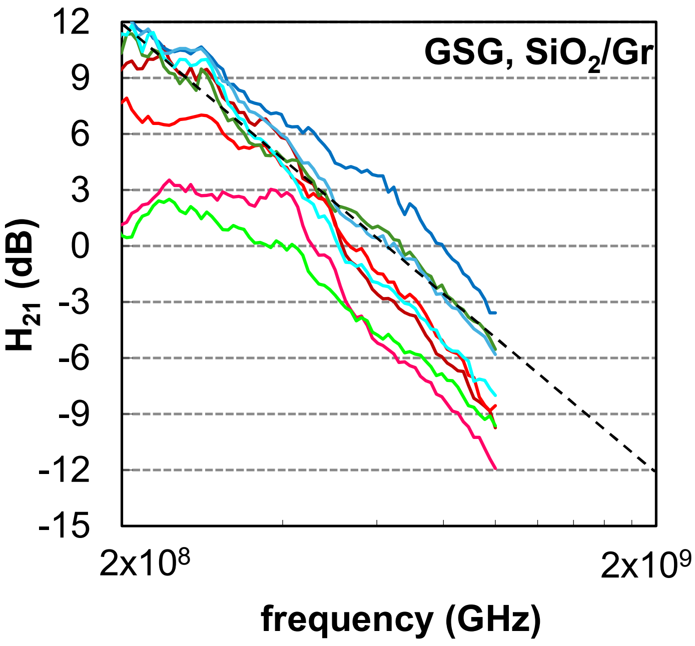

Figure S 7: H_{21} current gain as a function of frequency with a logarithmic frequency axis. The gain roll-off is about -24 dB/dec.

RAMAN MEASUREMENT OF MONOLAYER MoS_2

Figure S 8 shows the Raman spectrum of monolayer MoS_2 wet-transferred onto graphene. The MoS_2 modes E at 384-386 cm^{-1} and A_1 at 403-405 cm^{-1} are clearly visible. The E-A_1 peak distance was in the range of 18-20 cm^{-1} indicating the presence of monolayer MoS_2 with a thickness of around 0.7 nm. The D, G, and 2D modes of the underlying graphene are also visible in the Raman spectrum.

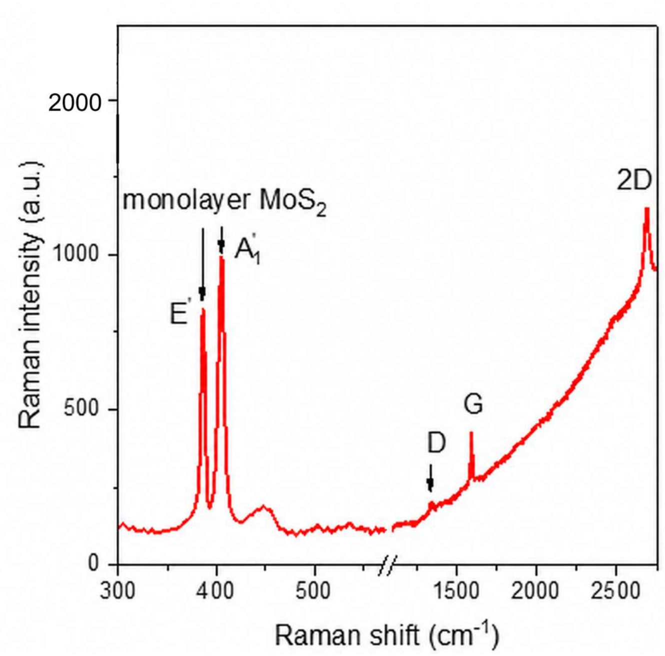

Figure S 8: Raman spectrum of monolayer MoS_2 transferred on Graphene.

(1) Lim, E. W.; Ismail, R. Conduction Mechanism of Valence Change Resistive Switching Memory: A Survey. *Electronics* **2015**, *4* (3), 586–613. https://doi.org/10.3390/electronics4030586.
